# Propensity Score Matching Analysis of Extracorporeal Versus Intracorporeal Anastomosis in Laparoscopic Colectomy for Right Colon Cancer

**DOI:** 10.7759/cureus.92010

**Published:** 2025-09-10

**Authors:** Duong V Hai, Vo C Nguyen, Tran Vinh Hung, Huu Nguyen, Giao H Quy, Phuc H Nguyen

**Affiliations:** 1 General Surgery, University of Medicine Center of Ho Chi Minh City, Ho Chi Minh City, VNM; 2 General Surgery, Pham Ngoc Thach University of Medicine, Binh Dan Hospital, Ho Chi Minh City, VNM; 3 Surgery, Tan Tao University, Ho Chi Minh City, VNM; 4 Surgery, Binh Dan Hospital, Ho Chi Minh City, VNM; 5 Gastrointestinal Surgery, Binh Dan Hospital, Ho Chi Minh City, VNM; 6 General Surgery, Bình Dan Hospital, Ho Chi Minh City, VNM; 7 Surgery, Pham Ngoc Thach University of Medicine, Ho Chi Minh City, VNM

**Keywords:** extracorporeal anastomosis, intracorporeal anastomosis, laparoscopic colectomy, propensity score matching analysis, right colon cancer

## Abstract

Background and objectives

In recent years, laparoscopic colectomy (LC) has become the standard of care for treating colon cancer worldwide. LC has two main techniques: laparoscopic-assisted colectomy with extracorporeal anastomosis (LAC/EA) and total LC with intracorporeal anastomosis (TLC/IA). Each technique has its advantages and disadvantages. Our study aimed to compare outcomes between intracorporeal anastomosis and EA in LC for right colon cancer.

Methods

This was a retrospective cohort study comparing 140 patients who underwent total LC with IA group) and 177 patients who underwent laparoscopic-assisted right colectomy with EA group, based on patients’ demographics and postoperative outcomes. Within postoperative outcomes, we also compared two subgroups: the extracorporeal hand-sewn (EHA) group (61 patients) and the extracorporeal stapler (ESA) group (116 patients) with the IA group. A propensity score matching analysis was performed to mitigate patient selection bias between the two groups and their subgroups.

Results

After propensity score matching, 131 patients in the EA group were compared with 131 in the IA group. In the subgroups, 107 patients in the ESA group and 56 in the EHA group were compared with the same number of patients in the IA group (107 and 56 patients, respectively). In the main groups, no statistically significant differences were observed in patients’ demographics, tumor location, cancer stage, or type of surgery.

The IA group had less intraoperative blood loss than the EA group (60.2 ± 29.9 ml vs. 72 ± 35.8 ml, p = 0.01), and the mean length of mini-laparotomy was shorter in the IA group (4.55 ± 0.75 cm vs. 6.21 ± 1.23 cm, p < 0.001). However, the mean operative time was significantly longer in the IA group (204 ± 38.5 min vs. 187 ± 41.8 min, p < 0.001). There was no significant difference in visual analogue scale (VAS) score (2.63 vs. 2.73, p = 0.22), time to flatus (2.53 vs. 2.75 days, p = 0.1), postoperative length of stay (7.05 vs. 7.44 days, p = 0.7), or rate of postoperative complications (7.6% vs. 13.7%, p = 0.1). The results of comparing the EHA and ESA groups with the IA group were similar.

Conclusion

Propensity score matching analysis revealed that the TLC/IA technique was superior to LAC/EA in terms of intraoperative blood loss and length of mini-laparotomy, although operative time was longer. Other outcomes, such as VAS, length of stay, time to flatus, and complication rate, showed no significant differences. Further randomized studies are required to draw definitive conclusions about the best anastomotic technique during laparoscopic right colectomy.

## Introduction

Until now, with many advances in treatment, chemotherapy and radiotherapy combined with surgery have improved the long-term outcomes of cancer treatment. However, surgery still plays a dominant role in the treatment of colon cancer. At many major surgical centers, laparoscopic colectomy is considered the standard treatment for curative colon cancer [1]. Many studies have demonstrated the significant advantages of laparoscopic surgery over open surgery in both short-term and long-term outcomes, as well as in terms of safety and oncological benefits. There are two types of anastomosis in laparoscopic colectomy: extracorporeal anastomosis (EA) and intracorporeal anastomosis (IA). Choosing between these two methods remains a consideration for many surgeons. EA is performed more frequently due to its lower technical requirements, lower costs, and shorter surgery times compared with IA [2]. However, EA requires greater mobilization of the colon and ileum, and the midline incision must be longer to facilitate subsequent steps, which can damage the mesentery due to tension and affect bowel function recovery [3]. Meanwhile, IA, considered more challenging and requiring a well-trained and experienced surgeon, can address the disadvantages of EA while ensuring compliance with oncological principles, allowing for the harvesting of more lymph nodes, avoiding exposure of the anastomosis to the external environment, and is gradually being adopted by more surgeons.

The present propensity score-matched (PSM) analysis aims to compare surgical outcomes in patients who underwent laparoscopic right colectomy with EA or IA for adenocarcinoma. Furthermore, we also compared the two EA subgroups, extracorporeal hand-sewn anastomosis (EHA) and extracorporeal stapler anastomosis (ESA), with the IA group.

## Materials and methods

Study design and patients

This was a non-randomized, retrospective study conducted at Binh Dan Hospital, Vietnam. We compared two selected patient groups and used propensity score matching (PSM) to minimize confounding bias inherent in a non-randomized study.

Inclusion Criteria

Inclusion criteria included histologically confirmed colonic adenocarcinoma diagnosed between April 2020 and April 2024, with tumour stage cT2-4, any N, and requiring right or right extended colectomy. Tumour stage was based on colonoscopy and preoperative CT scan findings.

Exclusion Criteria

Exclusion criteria were recurrent colorectal cancer or concomitant malignancies. Cases with distant metastases were also excluded. Additionally, patients undergoing colectomy without anastomosis were excluded.

Surgical procedure and postoperative management

Patients with tumors located in the cecum and ascending colon underwent laparoscopic right colectomy, while those with tumors in the hepatic flexure or proximal transverse colon near the hepatic flexure underwent laparoscopic extended right colectomy. All cases adhered to the principles of complete mesocolic excision (CME), and anastomoses were performed either intracorporeally or extracorporeally.

The decision to perform IA or EA was made at the discretion of the operating surgeon. In practice, IA was generally chosen by surgeons with greater laparoscopic experience and the availability of appropriate stapling devices, while EA was more commonly used in earlier cases or when the surgeon considered it technically more straightforward. No fixed patient-related criteria were applied for this choice.

We performed vascular pedicle dissection in accordance with CME principles, ensuring an intact mesocolic envelope.

EA

At this stage, a midline mini-laparotomy was created, and the bowel was exteriorized to complete mesenteric division and bowel transection/excision. For hand-sewn reconstruction, an end-to-end anastomosis was fashioned in one or two layers at the operating surgeon’s discretion. For stapled reconstruction, an isoperistaltic side-to-side anastomosis was performed: two linear stapler firings were used to transect the proximal and distal bowel, followed by a single linear stapler firing to create the side-to-side anastomosis. The common enterotomy was then closed with hand sutures. The mesenteric defect was closed through the midline incision.

IA

The mesentery was divided up to the bowel wall, and both bowel ends were transected using linear staplers. An isoperistaltic side-to-side anastomosis was then created with a single linear stapler firing. The common enterotomy and the mesenteric defect were closed laparoscopically. The specimen was retrieved through a short midline extraction incision.

Procedures were performed by four surgical teams. Each lead surgeon had at least 15 years of experience performing standard laparoscopic colectomies.

Cases were identified via retrospective chart review. Those meeting predefined eligibility criteria were included. Data were extracted using a standardized case report form by trained abstractors who were blinded to the comparative objectives of the study.

Postoperative pain was assessed on postoperative day 1 using a visual analog scale (VAS).

Anastomotic leak was diagnosed and graded according to the International Study Group of Rectal Cancer (ISREC) consensus definition (Grades A-C) [4].

Surgical site infection (SSI) was defined per the CDC criteria [5].

Postoperative ileus was diagnosed according to established criteria for prolonged postoperative ileus (PPOI) [6].

During the postoperative hospital stay, all patients received standardized care. Specifically, the nasogastric tube and urinary catheter were removed on the morning after surgery. On postoperative day 1, patients were provided parenteral nutrition and allowed to drink glucose water, progressing to oral intake once bowel function returned (evidenced by passing gas). Discharge criteria included the absence of abnormal symptoms, the ability to eat at least three meals per day, and successful bowel movements. The follow-up period for patients extended from the time of surgery to 30 days postoperatively.

Comparison criteria

Preoperative variables were used to compare the homogeneity between the two surgical groups. Intraoperative and postoperative parameters were analyzed to evaluate the safety and efficacy of the two groups.

Subgroup analysis was also performed. Specifically, we compared patients undergoing IA with those undergoing EA, categorized as hand-sewn (1) or stapled (2).

Statistical analysis

Data were analyzed using IBM SPSS Statistics for Windows, Version 26 (IBM Corp., Armonk, NY) and R Version 4.4 (R Foundation for Statistical Computing, Vienna, Austria; https://www.R-project.org/.

Continuous variables are expressed as mean ± SD, and categorical variables as frequencies and percentages. The nonparametric Mann-Whitney U test and the Chi-squared or Fisher’s exact test were used to compare continuous and categorical variables, respectively. A p-value < 0.05 was considered statistically significant.

PSM

To minimize the risk of bias and potential confounding factors and improve the validity of statistical comparisons, PSM was performed to match patients in the IA and EA groups at a 1:1 ratio, using the following covariates that might affect perioperative and postoperative outcomes: gender, age, BMI, tumor location, hemoglobin level, albumin level, and ASA grade.

**Figure 1:**
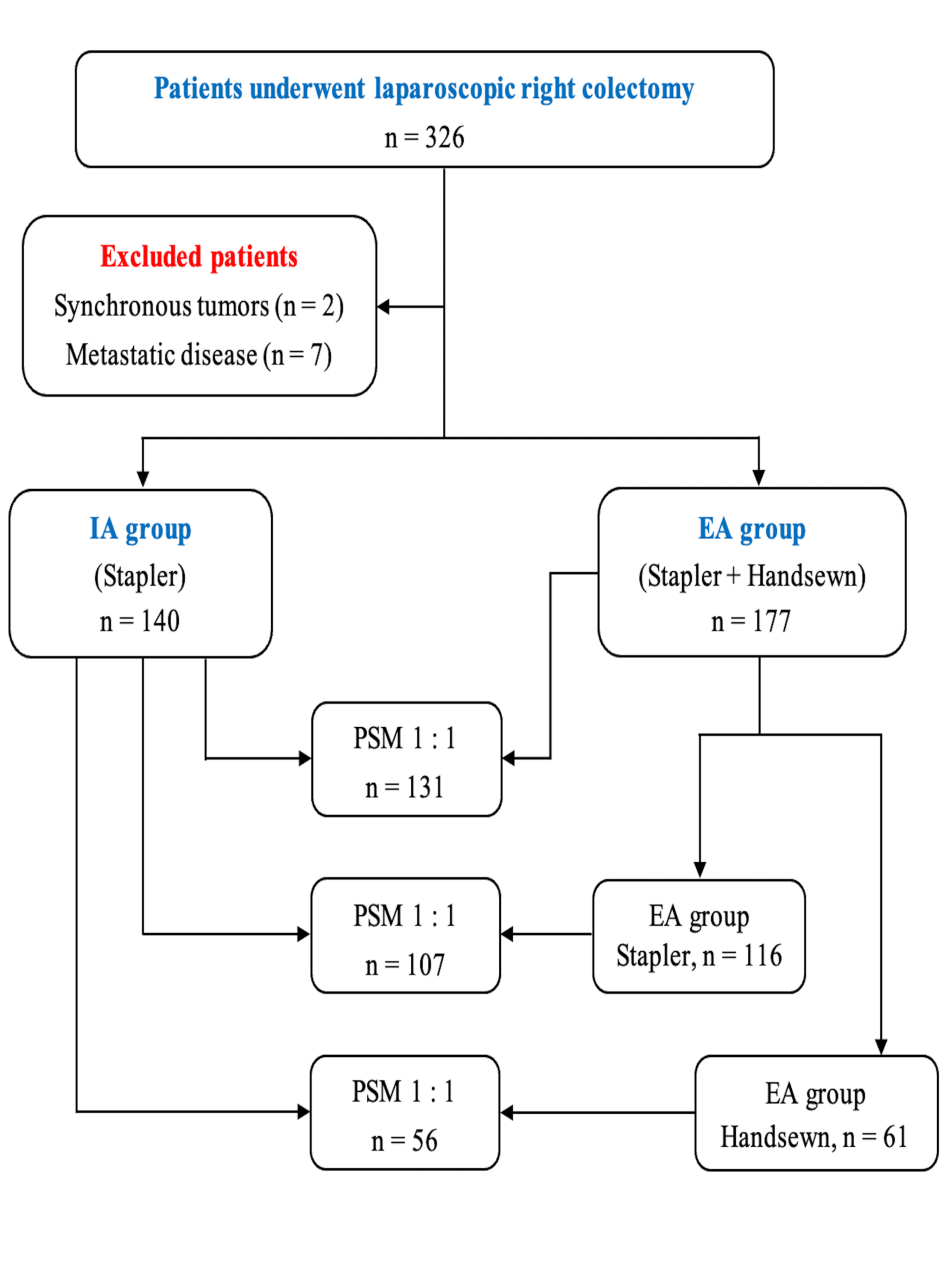
Flow diagram of the study.

**Figure 2:**
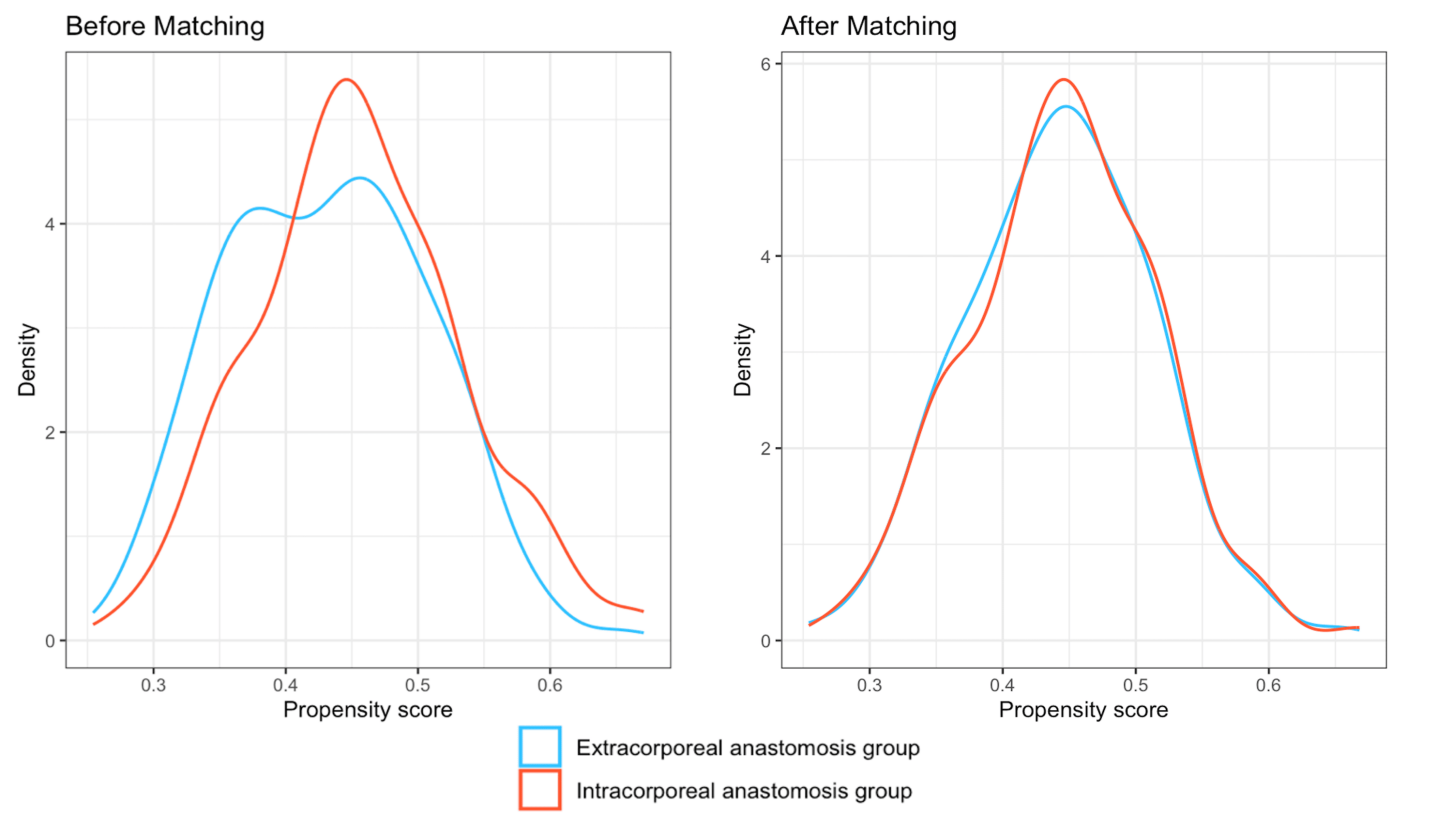
Propensity score before and after matching.

We used R software to generate propensity score values using the logistic regression method. The IA and EA groups were then paired 1:1 on these propensity scores. Nearest-neighbor matching was performed without replacement, at a ratio of 1:1, with a caliper of 0.2 SDs of the estimated logit (package MatchIt) (Figures 1-2).

## Results

After the PSM analysis, 131 of 177 EA group patients were matched with 131 of 140 IA group patients. The demographic variables and preoperative status are shown in Table 1. Before and after PSM, there were no significant differences in BMI, ASA classification, comorbidities, age, sex, pathological depth of tumor invasion, lymph nodes harvested, TNM stage, tumor location, type of procedure, and preoperative hemoglobin and albumin blood tests.

**Table 1:** Patient characteristics. * T-test ** Wilcoxon rank-sum test # Chi-square test ## Fisher’s exact test EA = stapler or hand-sewn; IA = stapler. EA: Extracorporeal anastomosis; IA: Intracorporeal anastomosis; ASA: American Society of Anesthesiologists.

Variable	Before matching				After matching			
	EA (N = 177)	IA (N = 140)	P-value	Test statistic	EA (N = 131)	IA (N = 131)	P-value	Test statistic
Age (years)	58.7 ± 12.6	59.4 ± 13.7	0.76**	W = 12142	58.8 ± 12.8	59.3 ± 13.6	0.891**	W = 8496
Gender (Female/Male)	46.9% / 53.1%	50.0% / 50.0%	0.582#	χ² = 0.30228	53.4% / 46.6%	48.9% / 51.1%	0.458#	χ² = 0.54991
BMI (kg/m²)	21.6 ± 1.96	21.8 ± 1.97	0.379**	W = 11677	21.6 ± 1.84	21.8 ± 1.96	0.674**	W = 8322
Comorbidities	29.90%	27.10%	0.584#	χ² = 0.29959	31.30%	27.50%	0.498#	χ² = 0.45981
ASA								
I	9 (5.1%)	8 (5.7%)	0.687#	χ² = 0.75122	9 (6.9%)	7 (5.3%)	0.765#	χ² = 0.53623
II	134 (75.7%)	100 (71.4%)			91 (69.5%)	96 (73.3%)		
III	34 (19.2%)	32 (22.9%)			31 (23.7%)	28 (21.4%)		
Albumin (g/L)	36.2 ± 3.46	36.8 ± 3.77	0.113**	W = 11109	36.3 ± 3.61	36.5 ± 3.65	0.659*	t = -0.4424
Hemoglobin (g/dL)	11.7 ± 1.41	11.9 ± 1.47	0.185**	W = 11316	11.7 ± 1.46	11.8 ± 1.38	0.688**	W = 8333.5
Procedure								
Right colectomy	100 (56.5%)	81 (57.9%)	0.808#	χ² = 0.05902	74 (56.5%)	72 (55.0%)	0.804#	χ² = 0.06188
Right extended colectomy	77 (43.5%)	59 (42.1%)			57 (43.5%)	59 (45.0%)		

Outcomes

Before PSM, the EA group had a shorter operative time (187 ± 40 mins vs. 205 ± 38.2 mins, p < 0.001), but experienced higher intraoperative blood loss (74 ± 38.6 ml vs. 58.9 ml, p < 0.001), and a longer mean length of mini-laparotomy (6.32 ± 1.09 cm vs. 6.21 ± 1.23 cm, p < 0.001) compared to the IA group. Recovery after surgery was better for patients who underwent IA, as confirmed by a shorter time to flatus (2.52 ± 0.844 days vs. 2.78 ± 0.943 days, p = 0.016), reduced postoperative pain indicated by the mean VAS Scale (2.66 ± 0.665 vs. 2.85 ± 0.75, p < 0.02), and a lower rate of postoperative complications (7.9% - 11 cases vs. 16.4% - 29 cases, p = 0.023). The wound infection rate was also statistically higher in the EA group (9.6% - 17 cases vs. 1.4% - 2 cases, p = 0.0023). At the same time, other complications, including ileus, anastomotic leakage, and anastomotic bleeding, were similar between the two groups. Furthermore, there were no significant differences in postoperative length of stay (7.06 ± 1.02 days vs. 7.42 ± 2.62 days, p = 0.46).

After PSM, the IA group still had a longer operative time (204 ± 38.5 mins vs. 187 ± 41.8 mins, p < 0.001) but lower intraoperative blood loss (60.2 ± 29.9 ml vs. 72 ± 35.8 ml, p = 0.01). The mean length of mini-laparotomy was also shorter (4.55 ± 0.746 cm vs. 6.11 ± 1.13 cm, p < 0.001). However, the time to flatus (2.53 ± 0.826 days vs. 2.75 ± 0.979 days, p = 0.1), mean VAS score (2.63 ± 0.637 vs. 2.73 ± 0.69, p = 0.227), postoperative length of stay (7.05 ± 1.05 days vs. 7.44 ± 2.95 days, p = 0.73), and the rate of postoperative complications (7.6% - 10 cases vs. 13.7% - 18 cases, p = 0.11) remained comparable. However, the wound infection rate was still higher in the EA group (7.6% - 10 cases vs. 1.5% - 2 cases, p = 0.0018) (Table 2).

**Table 2:** Operative variables and early postoperative outcomes. * T-test ** Wilcoxon rank-sum test # Chi-square test ## Fisher’s exact test EA = stapler + hand-sewn; IA = stapler. EA: Extracorporeal anastomosis; IA: Intracorporeal anastomosis; VAS: Visual Analogue Scale

Variable	Before matching				After matching			
	EA (N = 177)	IA (N = 140)	P-value	Test statistic	EA (N = 131)	IA (N = 131)	P-value	Test statistic
Operative time (min)	187 ± 40.0	205 ± 38.2	< 0.001**	W = 9169	187 ± 41.8	204 ± 38.5	< 0.001**	W = 6386.5
Blood loss (ml)	74.0 ± 36.8	58.9 ± 29.9	< 0.001**	W = 15344	72.0 ± 35.8	60.2 ± 29.9	0.01**	W = 10148
Incision length (cm)	6.32 ± 1.17	4.55 ± 0.77	< 0.001**	W = 21966	6.11 ± 1.13	4.55 ± 0.75	< 0.001**	W = 14854
VAS	2.85 ± 0.75	2.66 ± 0.67	0.0229**	W = 14079	2.73 ± 0.69	2.63 ± 0.64	0.227**	W = 9250.5
Time to flatus (days)	2.78 ± 0.94	2.52 ± 0.84	0.0164**	W = 14174	2.75 ± 0.98	2.53 ± 0.83	0.103**	W = 9495.5
Hospital stay (days)	7.42 ± 2.62	7.06 ± 1.02	0.469**	W = 12901	7.44 ± 2.95	7.05 ± 1.05	0.732**	W = 8765.5
Ileus	11 (6.2%)	5 (3.6%)	0.286#	χ² = 1.1396	8 (6.1%)	4 (3.1%)	0.237#	χ² = 1.3973
Anastomotic leak	4 (2.3%)	1 (0.7%)	0.388##	Fisher’s exact test	4 (3.1%)	1 (0.8%)	0.37##	Fisher’s exact test
Anastomotic bleeding	4 (2.3%)	3 (2.1%)	1##	Fisher’s exact test	2 (1.5%)	3 (2.3%)	1##	Fisher’s exact test
Wound infection	17 (9.6%)	2 (1.4%)	0.0023#	χ² = 9.274	10 (7.6%)	2 (1.5%)	0.0181#	χ² = 5.5893
Overall complications	29 (16.4%)	11 (7.9%)	0.0232#	χ² = 5.1549	18 (13.7%)	10 (7.6%)	0.11#	χ² = 2.5592

In subgroups, we compared 107 of 116 patients in the ESA group with 107 of 140 patients in the IA group, and 56 of 61 patients in the EHA group with 56 of 140 patients in the IA group (after PSM analysis). We noticed that the ESA group had a shorter operative time (183 mins vs. 207 mins, p < 0.001) and a longer mean incision length (6.07 cm vs. 4.55 cm, p < 0.001); however, there was no significant difference in intraoperative blood loss between the two groups (68.7 ml vs. 61 ml, p = 0.1). Meanwhile, the EHA group also had a longer incision length (6.73 cm vs. 4.48 cm, p < 0.001). Still, the operative time (193 mins vs. 205 mins, p = 0.1) and intraoperative blood loss (72.4 ml vs. 63.9 ml, p = 0.06) showed no significant differences. Postoperative outcomes did not reveal any statistically significant differences between the ESA and EHA groups compared to the IA group, such as time to flatus, VAS score, and postoperative length of stay (Table 3). The ESA group had three anastomotic leakage cases, the EHA group had one leakage case, and the IA group had one case in both comparisons. The wound infection rate was lower in the EHA group (14.3% vs. 1.8%, p = 0.03), but there was no significant difference when comparing the ESA and IA groups (6.5% vs. 1.9%, p = 0.17) (Table 3).​​​​​​​

**Table 3:** Operative variables and early postoperative outcomes in subgroups. *T-test ** Wilcoxon rank-sum test # Chi-square test ##  Fisher’s exact test ESA: Extracorporeal stapler; IA: Intracorporeal anastomosis; EHA: Extracorporeal hand-sewn; VAS: Visual analogue scale.

Subgroup	After matching				After matching			
	ESA (Stapler) (N=107)	IA (Stapler) (N=107)	P-value	Test statistic value	EHA (Hand-sewn) (N=56)	IA (Stapler) (N=56)	P-value	Test statistic value
Operative time (min)	183 ± 42.4	207 ± 38.8	<0.001**	W = 3795.5	193 ± 35.6	205 ± 39.4	0.106**	W = 1290.5
Blood loss (ml)	68.7 ± 33.6	61.0 ± 30.3	0.109**	W = 6428	72.4 ± 28.7	63.9 ± 32.0	0.062**	W = 1877
Incision length (cm)	6.07 ± 1.13	4.55 ± 0.755	<0.001**	W = 9811.5	6.73 ± 1.14	4.48 ± 0.81	<0.001**	W = 2946
VAS	2.79 ± 0.75	2.64 ± 0.64	0.15**	W = 6315	2.82 ± 0.69	2.66 ± 0.721	0.235**	W = 1755.5
Time to flatus (days)	2.74 ± 0.99	2.56 ± 0.913	0.27**	W = 6183	2.89 ± 0.93	2.64 ± 0.99	0.134**	W = 1808.5
Hospital stay (days)	7.49 ± 3.22	6.99 ± 0.906	0.99**	W = 5728	7.41 ± 1.36	7.18 ± 1.22	0.143**	W = 1793.5
Ileus	7 (6.5%)	5 (4.7%)	0.552#	χ² = 0.353	4 (7.1%)	3 (5.4%)	1##	Fisher’s exact test
Anastomotic leak	3 (2.8%)	1 (0.9%)	0.621##	Fisher’s exact test	1 (1.8%)	1 (1.8%)	1##	Fisher’s exact test
Anastomotic bleeding	2 (1.9%)	2 (1.9%)	1##	Fisher’s exact test	2 (3.6%)	3 (5.4%)	1##	Fisher’s exact test
Wound infection	7 (6.5%)	2 (1.9%)	0.17##	Fisher’s exact test	8 (14.3%)	1 (1.8%)	0.032##	Fisher’s exact test
Overall complications	13 (12.1%)	10 (9.3%)	0.508#	χ² = 0.438	14 (25.0%)	8 (14.3%)	0.154#	χ² = 2.036

## Discussion

Both before and after PSM, the IA group had a longer operative time than the EA group, due to the additional time required to perform IA. This result is consistent with findings from other authors and may be explained by the fact that performing IA requires resecting the mesentery, positioning the two ends of the colon correctly, closing the enterotomy after stapling, and closing the mesenteric defect entirely laparoscopically. Suturing inside the abdominal cavity via laparoscopy is a challenging and time-consuming procedure, particularly for surgeons who have not yet achieved the learning curve [7].

The amount of blood lost during surgery not only reflects the short-term safety of the surgical method but is also one of the factors affecting long-term outcomes of surgery for gastrointestinal cancer [8]. After PSM, the average blood loss in the IA group was 60.2 ml, which was statistically lower (p = 0.01) compared to the EA group (72 ml). Wu Q et al. [9] analyzed 15 articles and 4 abstracts with a total of 1957 patients undergoing colectomy. Intraoperative blood loss varied by study, with an average of 31.2-105 ml in the IA group and 43.3-231.3 ml in the EA group (p = 0.03). The reason for this difference is that pulling out the colon in the EA group may cause the mesentery to stretch, increasing the risk of mesenteric tearing and bleeding. In our study, one patient had intraoperative blood loss of 400 ml. During extracorporeal anastomosis, there was tension on the transverse colon mesentery, and the mesentery was torn along with a blood vessel, which led to significant bleeding and extension of the surgical incision to control the hemorrhage.

In the EA group, the incision is usually in the midline and must be wide enough for colon resection and anastomosis, whereas in the IA group, the incision is used only to retrieve the specimen. This incision can be placed in different positions, such as the Pfannenstiel incision. Furthermore, the incision can be shorter than the tumor size, which is nearly impossible with the EA group. Zhang H et al. [2] compiled five RCT studies involving 559 patients, and in the IA group, more than half of the patients (63.7%) underwent surgery using the Pfannenstiel incision. The rates of surgical site hernia and postoperative pain in this group were lower due to fewer muscle layers being sutured, shorter operation time, and shorter incision length [10,11].

van Oostendorp S et al. report indicates that mid/upper abdominal incisions tend to be more painful and significantly affect respiratory function compared to lower incisions such as Pfannenstiel [12]. Bollo J et al.'s randomized clinical trial, with 140 patients undergoing right hemicolectomy for cancer, assessed postoperative pain over five days. The study revealed mean pain scores ranging from 1.71 to 2.73 for the IA group and 2.36 to 3.01 for the extracorporeal group (p = 0.035) [13]. Analgesic dosage was also lower (p = 0.001) in the IA group compared to the extracorporeal group, correlating with statistically shorter mean incision lengths in the intracorporeal group (p < 0.001).

The time to flatus is an indicator of bowel motility recovery after surgery. In our study, the IA group had a shorter time to flatus compared to the EA, EHA, and ESA groups; however, after PSM, this difference was no longer significant. Antonio Biondi explains that this difference is due to tension on the mesentery and bowel in the EA group when pulling out the colon, leading to slower bowel function recovery compared to the IA group [14].

According to our results, after PSM, no significant difference was observed in the overall postoperative complication rates among the three comparative groups. When individual complication types were analyzed, only wound infection rates demonstrated a statistically significant difference (the IA group had a lower rate of wound infection compared to the EA and EHA groups). Studies indicate that wound infection occurs in 1%-30% of colorectal surgeries [15,16]. Colorectal surgery itself is a high-risk factor for intra-abdominal and wound infection; however, laparoscopic surgery significantly reduces the incidence of SSIs in these patients [17].

There were no significant differences in anastomotic leakage rates between IA and EA or IA and the two subgroups. Some researchers suggest that anastomotic leakage in the IA group may cause more severe clinical consequences due to greater anastomotic disruption compared to the EA group [18]. Espin E et al. conducted an analysis involving 116 patients who experienced anastomotic leakage after right hemicolectomy [19]. They categorized leakages based on the anastomosis type (handsewn vs. stapler) and severity according to the Clavien-Dindo classification and the International Study Group of Rectal Cancer (ISGRC) criteria. Their findings indicated that Clavien-Dindo grade III-a leakages were more common in patients who had a handsewn anastomosis, while grade III-b leakages were more frequent in those with a stapled anastomosis. Likewise, ISGRC grade C leakage was more prevalent in patients with stapled anastomosis. Additional studies are needed to draw definitive conclusions on the severity of postoperative anastomotic leakage in relation to the type of anastomosis. The treatment required, including reoperation and readmission, should be considered as endpoints when comparing EA and IA [18].

The restoration of bowel activity (expressed as time to flatus and time to stool) and postoperative complications are important factors affecting postoperative length of stay. In our study, all groups had a similar rate of postoperative complications and time to flatus, so the lack of difference in length of stay is reasonable.

This study carries the inherent limitations of any retrospective analysis. In particular, the choice between intracorporeal and extracorporeal anastomosis was not randomized but was based on the surgeon’s preference and experience. This inherent variability in surgical decision-making could introduce selection bias. To minimize this limitation, we applied PSM using covariates (age, sex, BMI, tumor location, hemoglobin, albumin, ASA grade). We confirmed that all post-match standardized mean differences were <0.1, indicating adequate balance between groups.

However, our propensity score model did not incorporate several other potentially relevant factors, such as diabetes mellitus, smoking status, steroid or immunosuppressive use, prior abdominal surgery, tumor size, emergency versus elective setting, extraction site location, drain placement, or adherence to ERAS protocols. These unmeasured covariates may have influenced both surgical technique selection and postoperative outcomes, and their omission may affect the completeness of the matching process.

In addition, our study was conducted at a single institution, limiting the ability to assess differences in surgical techniques and decision-making across centers. Finally, the follow-up period was limited to 30 days, and longer-term oncological and functional outcomes were not evaluated.

Another limitation is that postoperative pain was assessed only once, on day 1, using the VAS scale. This approach provides only an early snapshot of pain and may not reflect the full postoperative trajectory. A more comprehensive evaluation with serial assessments and analgesic consumption would better capture the true pain burden after surgery.

Despite these limitations, our results provide clinically relevant insights and support the use of PSM as a robust method to reduce bias when randomized controlled trials are not feasible.

Therefore, while our findings are informative, they should be interpreted with caution and ideally confirmed in well-designed, multicenter, randomized controlled trials.

## Conclusions

Propensity-score matching analysis revealed that the TLC/IA technique was superior to the LAC/EA technique in terms of intraoperative blood loss and length of mini-laparotomy, although the operative time was longer. Other outcomes, such as VAS, length of stay, time to flatus, and complication rate, showed no significant differences. Further multicenter randomized studies with longer follow-up are required to draw definitive conclusions regarding the optimal anastomotic technique during laparoscopic right colectomy.
